# Supplementary material for: Establishment of an Integrated CRISPR/Cas9 Plasmid System for Simple and Efficient Genome Editing in Medaka In Vitro and In Vivo
Source: Biology (Basel). 2023 Feb 20;12(2):336. doi: 10.3390/biology12020336 (PMC9953409; doi:10.3390/biology12020336)
Supplement: Supplementary file 1 [file biology-12-00336-s001.zip › biology-2182740-supplementary.pdf]

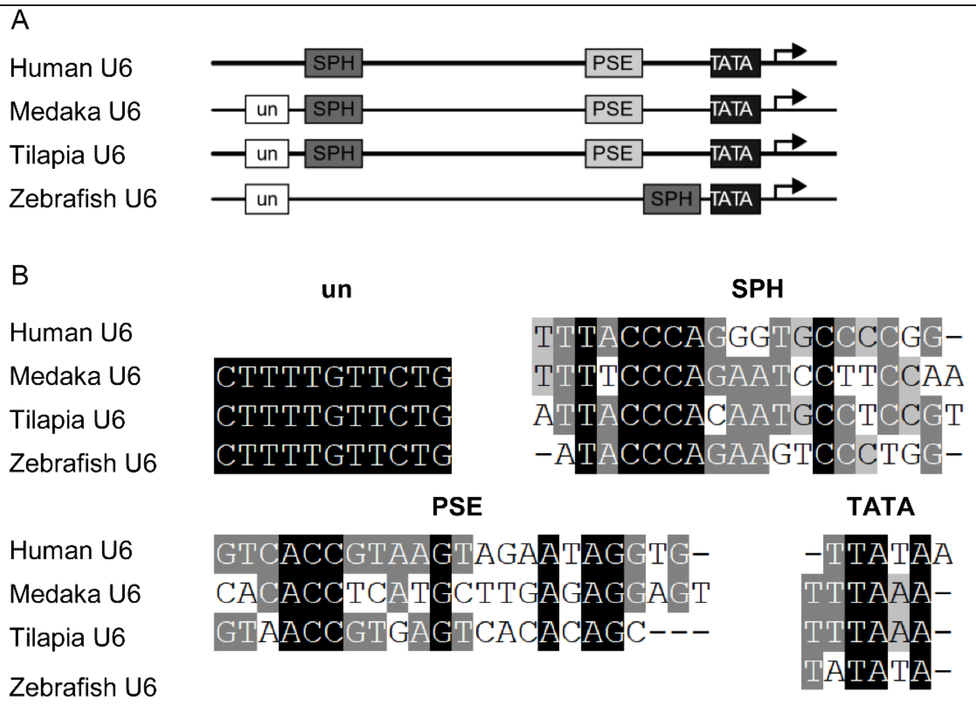

Figure S1. Analysis of U6 promoters from human and different fishes. (**A**) Diagram of sequence alignment of U6 promoters from human, medaka, Nile tilapia and zebrafish. (**B**) Nucleotide sequence of homology elements. un, an uncharacterized consensus; SPH, SphI postoctamer homology elements; PSE, proximal sequence elements; TATA, TATA box).

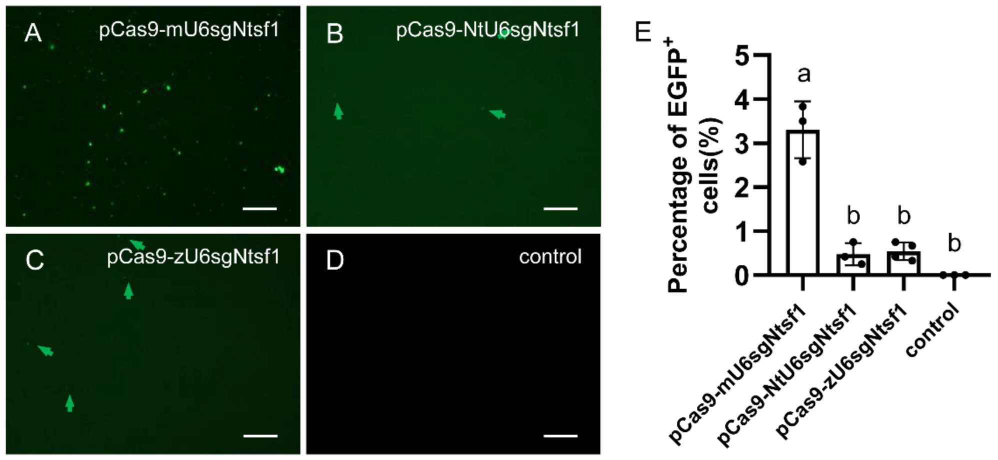

Figure S2. Monitoring the gene editing efficacy of pCas9-U6sgRNA in MES1 with pGNtsf1 reporter vector 48 h after transfection. (**A**–**D**) Co-transfection of pCas9-U6sgRNA targeting Nile tilapia *sf1* and pGNtsf1 in SG3. The sgRNA targeting Nile tilapia *sf1* in pCas9-U6sgRNA is driven by U6 promoters derived from medaka (pCas9-mU6sgNtsf1) (**A**), Nile tilapia (pCas9-NtU6sgNtsf1) (**B**) and zebrafish (pCas9-zU6sgNtsf1) (**C**), respectively. SG3 cells transfected only with pGNtsf1 were set as negative control (**D**). (Scale bars, 100 μm). (**E**) Quantification of the percentage of EGFP positive cells in different groups. Data are shown as means ± standard derivation. Different letters above the bars represent significant differences between the groups ($p < 0.05$).

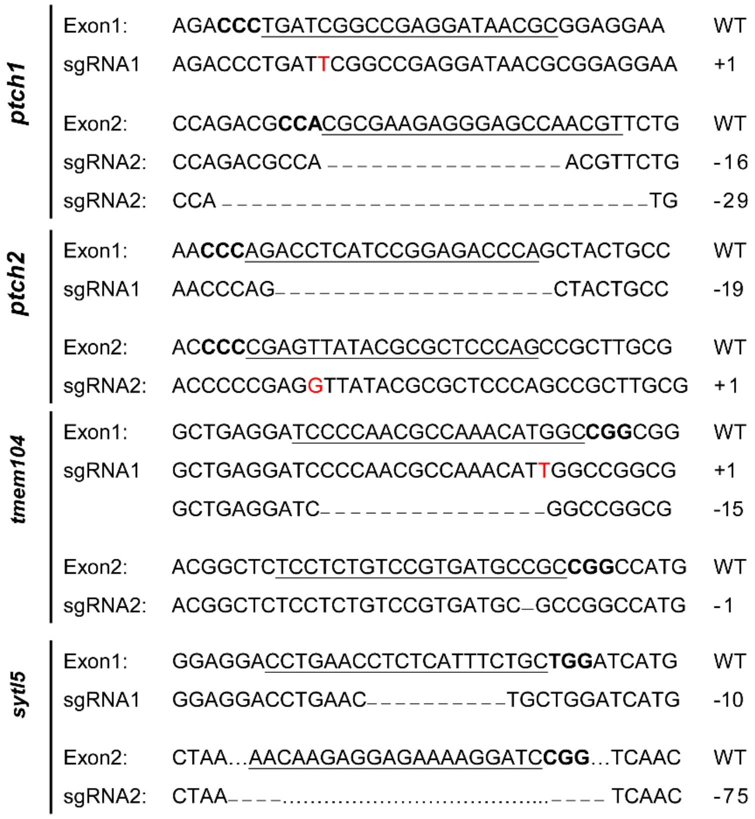

Figure S3. Target site design and mutation type detection of endogenous genes *ptch1, ptch2, sytl5* and *tmem104* in medaka cultured cells. The sgRNA target sites are indicated by underline and PAM sequences are indicated in bold. PCR amplicons of genomic DNA from each group were sub-cloned into plasmid followed by sequencing and sequencing results indicating different mutations were listed. Base insertions are shown in red font, and base deletions are shown in dashes.

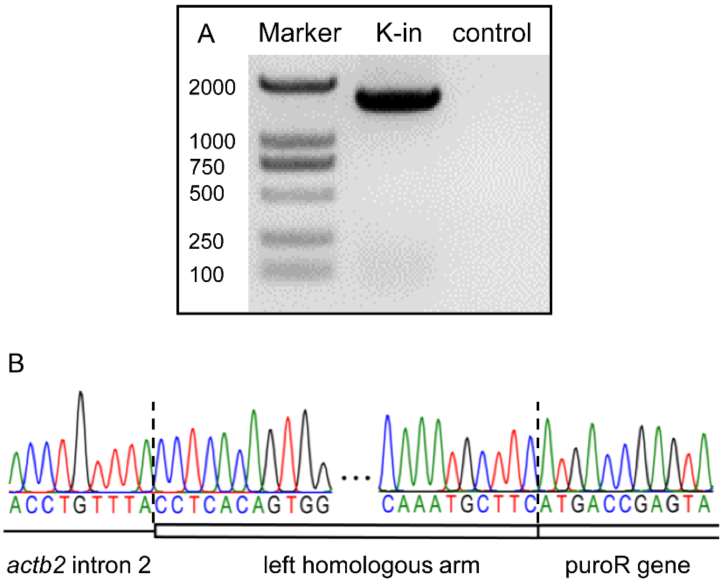

Figure S4. (**A**) Knock-in detection by specific primer pair. Knock-in detection primer pair was used to amplify corresponding genomic DNA. Amplicon was separated with agarose gel electrophoresis. K-in lane was amplicon from genomic DNA of pCas9-mU6sgactb2 and donor plasmid co-transfected SG3 cells. Control lane was amplicons from DNA of wild-type genome. (**B**) Sequencing chromatogram of amplicon of genomic DNA from pCas9-mU6sgactb2 and donor plasmid co-transfected SG3 cells. Result shown the correct conjunction between actb2 intron 2, left homologous arm, and puromycin resistance gene. puroR, puromycin resistance gene.

Table S1. Sequences of the primers used in this study.

Usage	Primer Names	Primer Sequence (5′-3′)
Gene editing plasmid construction	U6Mlu-F	CTTGACGAGTTCTTCTGAACGCGTCTCGAGCCTCTAGA
	scaffoldSal-R	AATTGGCGGTCGACTGGCGTAATAGCCAAC
	mU6sgptch1-R1	TGATCGGCCGAGGATAACGCCGATGAGCCAAAGTCTCTGAG
	sgptch1-F1	GCGTTATCCTCGGCCGATCAGTTTTAGAGCTAGAAATAGCAAGTTAAAAT
	mU6sgptch1-R2	CGCGAAGAGGGAGCCAACGTCGATGAGCCAAAGTCTCTGAG
	sgptch1-F2	ACGTTGGCTCCCTCTTCGCGGTTTTAGAGCTAGAAATAGCAAGTTAAAAT
	mU6sgptch2-R1	AGACCTCATCCGGAGACCCACGATGAGCCAAAGTCTCTGAG
	sgptch2-F1	TGGGTCTCCGGATGAGGTCTGTTTTAGAGCTAGAAATAGCAAGTTAAAAT
	mU6sgptch2-R2	CGAGTTATACGCGCTCCCAGCGATGAGCCAAAGTCTCTGAG
	sgptch2-F2	CTGGGAGCGCGTATAACTCGGTTTTAGAGCTAGAAATAGCAAGTTAAAAT
	mU6sgtmem104-R1	GCCATGTTTGGCGTTGGGGACGATGAGCCAAAGTCTCTGAG
	sgtmem104-F1	TCCCCAACGCCAAACATGGCGTTTTAGAGCTAGAAATAGCAAGTTAAAAT
	mU6sgtmem104-R2	GCGGCATCACGGACAGAGGACGATGAGCCAAAGTCTCTGAG
	sgtmem104-F2	TCCTCTGTCCGTGATGCCGCGTTTTAGAGCTAGAAATAGCAAGTTAAAAT
	mU6sgsytl5-R1	GCAGAAATGAGAGGTTCAGGCGATGAGCCAAAGTCTCTGAG
	sgsytl5-F1	CCTGAACCTCTCATTTCTGCGTTTTAGAGCTAGAAATAGCAAGTTAAAAT
	mU6sgsytl5-R2	GATCCTTTTCTCCTCTTGTTCGATGAGCCAAAGTCTCTGAG
	sgsytl5-F2	AACAAGAGGAGAAAAGGATCGTTTTAGAGCTAGAAATAGCAAGTTAAAAT
	mU6sgsall4-R1	ACCGATGAATTCAGACCCTGCGATGAGCCAAAGTCTCTGAG
	sgsall4-F1	CAGGGTCTGAATTCATCGGTGTTTTAGAGCTAGAAATAGCAAGTTAAAAT
	mU6sgNtsf1-R1	CCCAGTACCAGTACACAGCCCGATGAGCCAAAGTCTCTGAGTG
	sgNtsf1-F1	GGCTGTGTACTGGTACTGGGGTTTTAGAGCTAGAAATAGCAAGTTAAAAT
Mutation detection	ptch1-TIDE-F1	AGCCGAAGACTCGTGTATGG
	ptch1-TIDE-R1	CAGGTTAACGCAGAAGCCAC
	ptch1-TIDE-F2	TTAATGGGGGATGCCAGTCA
	ptch1-TIDE-R2	TGGGAGTTCTCACCTGATCC
	ptch2-TIDE-F1/2	TCGGCGCATAATGTTGGGA
	ptch2-TIDE-R1/2	CTGTAACACGTCCAAGCATCC
	tmem104g1/2-TIDE-F	GTGTGGTTACATGCGAAGGTG
	tmem104g1/2-TIDE-R	CTGACGAGTGCAGAAGGTGA
	sytl5-TIDE-F1/2	GATCCTCGTAAGGCTGGTGT
	sytl5-TIDE-R1/2	CTCACAGAGTTCTCCCCGGTC
	ptch1-PAGE-F1	CTAATGCACCCTCCGAACAG
	ptch1-PAGE-R1	ATCGCAGTAACTCGGTCGCT
	ptch1-PAGE-F2	GGTGGGCGAGTAAACCAAGA
	ptch1-PAGE-R2	ACATGAACTCTACTGGCTCGC
	ptch2-PAGE-F1	TGGACTATGGCCTCGGATCG
	ptch2-PAGE-R1	GTTTAAGTGCGAAAGCAGCGT
	ptch2-PAGE-F2	CTTTGGACTATGGCCTCGGAT
	ptch2-PAGE-R2	CCGCATCACCTTGGATATCTGT
	tmem104-PAGE-F1/2	AACCTTCTCTCACACGGCAG
	tmem104-PAGE-F1/2	TCACAAACGGAGAGTACGGC
	sytl5-PAGE-F1/2	TTTACCCAAAGGAGCCAATGGA
	sytl5-PAGE-R1/2	GAAGCGTTGATGGAATGGTGA
Reporter plasmid construction	Ntsf1-GFP-F1	CCCAGTACCAGTACACAGCCCGAGGGCGAGGGCGATGCCA
	GFP-Ntsf1-R1	GGCTGTGTACTGGTACTGGGTGGCATCGCCCTCGCCCTCG
	GFP-F	GACCACCAGGGCAAGGGTCTG
	GFP-R	CCAAACTCATCAATGTATCTTATC

Knock-in detection	knockin-F	GCACCACACCTTCTACAATGAGC
	knockin-R	GACGCGCGTGAGGAAGAGTTC

Table S2. Candidate off-target sequences and corresponding detection primer pairs.

Usage	Primer Names	Primer Sequence (5′-3′)	Candidate off -Target Sequence (5′-3′)
Off-target detection	Ptch1gRNA1-off1-F1	ACATATGTGGCTCCATCGGTT	CCGCTCTCCTCGGCGGATCA**TGG**
	Ptch1gRNA1-off1-R1	ACACTTCTCAGGTCGGTTCAC	
	Ptch1gRNA2-off1-F1	GACCGACATCCCTACGAGGAC	AAGATGGCGCCCTCTTCTCG**AGG**
	Ptch1gRNA2-off1-R1	GTTAATCCAGCGTCAGCGGT	
	tmem104gRNA1-off1-F1	ACACAACATTAGTAAAACTGCAT	GCCTAAACGCCTAACATGGC**TGG**
	tmem104gRNA1-off1-R1	GAATTGATTAGCCCGGCCTATG	
	tmem104gRNA1-off2-F1	TCACAGAGCTGCCAGAGTAACAC	TGCCCAACGCCAGACATGGA**GGG**
	tmem104gRNA1-off2-R1	CCATTTATCCTGCCATTGAAGCT	
